# Exploring the effect of pre-clinical Alzheimer’s disease on blood pressure using Mendelian randomisation and parental dementia as an instrumental variable in UK Biobank

**DOI:** 10.1186/s12916-025-04295-5

**Published:** 2025-08-20

**Authors:** Jennifer C. Palmer, Emma Hart, Emma Anderson, Seth Love, Deborah A. Lawlor

**Affiliations:** 1 Bristol Medical School, Population Health Sciences, University of Bristol, Bristol, UK; 2 MRC Integrative Epidemiology Unit, University of Bristol, Bristol, UK; 3 School of Physiology, Pharmacology and Neuroscience, University of Bristol, Bristol, UK; 4 Dementia Research Group, Institute of Clinical Neurosciences, Bristol Medical School, University of Bristol, Bristol, UK

**Keywords:** Blood pressure, Pre-clinical Alzheimer’s disease, Prospective cohort, Instrumental variables, Human population

## Abstract

**Background:**

Evidence suggests there may be a bidirectional relationship between high blood pressure (BP) and Alzheimer’s disease (AD). It is hypothesised that this is due to cerebral changes during pre-clinical AD that cause elevation of systemic BP. We aimed to test this by exploring the effect of risk of pre-clinical AD on blood pressure.

**Methods:**

We used data from the UK Biobank, including adults without prevalent or incident (within first 5 years of follow-up) clinical AD (*N* = 501,420, mean age 56.6, SD 8 years). We used two instrumental variables, an age-weighted parental dementia instrument score and a participant genetic instrument score, that are vulnerable to differing biases, to instrument risk of pre-clinical AD (the exposure). We tested the association of both instrument scores with systolic BP (SBP), diastolic BP (DBP), and hypertension. Sensitivity analyses were undertaken to explore different biases.

**Results:**

Both the higher parental dementia instrument and participant genetic instrument score were associated with higher mean SBP (difference in mean SBP mmHg per 1SD higher score: 0.12, 95% CI 0.06 to 0.17, p < 0.0001, and 0.07, 95% CI 0.00 to 0.13, p=0.037, respectively) but not DBP. Sensitivity analyses were largely consistent with these findings.

**Conclusions:**

Our findings provide preliminary evidence that pre-clinical AD increases SBP. Further research is required to determine whether this increase in SBP is due to increased cerebrovascular resistance as a result of pre-clinical AD. Obtaining a better understanding of the changing relationship with BP at different stages of AD may enable effective optimisation and targeting of therapies.

**Supplementary Information:**

The online version contains supplementary material available at 10.1186/s12916-025-04295-5.

## Background

Alzheimer’s disease is often divided into three stages based on the clinical presentation: the pre-clinical stage, characterised by normal cognition; the prodromal stage, characterised by mild cognitive impairment; and the dementia stage, where there is functional cognitive impairment [1]. Although Alzheimer’s disease dementia usually occurs in later life, there are early disease processes in the brain, such as a decline in cerebral blood flow, that precede clinical presentation by up to 20 years before diagnosis [2–5]. 

Hypertension is the leading preventable risk factor for cardiovascular disease and mortality worldwide. Hypertension affects 1 in 3 adults worldwide, and globally nearly half of people with hypertension are unaware of their condition [6]. Often, the cause of elevated blood pressure (BP) is not identified, but it may stem from reduced perfusion to any of several major organs, namely kidney, lungs, and importantly, the brain [7].

Studies suggest a complex relation between blood pressure and Alzheimer’s disease. A number of observational retrospective and prospective cohort studies have observed that hypertension which develops during mid-life (approx. 40–65 years) is associated with increased risk of Alzheimer’s disease later in life [8–13]. Some studies have found that having high BP later in life (> 65 years) is associated with lower risk of Alzheimer’s disease [14, 15]. The results of these observational studies may be exaggerated by residual confounding. They may also be underestimated by survivor bias, given that those with higher BP from earlier in life are more likely to die in a given time period compared to those with lower BP, or those whose BP rises only in later life. We identified two randomised controlled trials (RCTs) that explored the relation of anti-hypertensives with Alzheimer’s disease. The first used data from a large RCT that was established to assess the effects of different classes of antihypertensives and statins on coronary heart disease. It was used to compare effects of different antihypertensives on Alzheimer’s disease or related diseases incidence by linking the trial data to 18 years of follow-up data passively collected from Medicare recorded diagnoses. It found no differences in the risk of Alzheimer’s disease across different antihypertensives. However, for our purpose, as each antihypertensive class will have reduced blood pressure by a similar amount, this does not provide evidence of the effect of lowering blood pressure on Alzheimer’s disease [16]. The second RCT compared the effect of randomisation to intensive systolic blood pressure (SBP) reduction (aiming to reduce it to < 120 mmHg) compared to randomisation to standard treatment at the time (aiming for a reduction of < 140 mmHg) amongst adults > 50 years with hypertension and found no evidence of a difference between the two groups in risk of adjudicated probable dementia. This large RCT that achieved important differences in SBP between the two randomised arms, suggested that greater lowering of SBP does not causally reduce dementia, though it was unable to determine the specific effect on Alzheimer’s disease [17]. Early termination meant participants were only followed up for < 8 years, with fewer than expected cases of dementia, so the trial may have been underpowered. Thus, it is unclear from observational and RCT studies whether there is a causal relationship between Alzheimer’s disease and blood pressure.

Instrumental variable analyses are an alternative to conventional multivariable regression that can be used in observational studies. They can provide causal estimates of effect if they adhere to three core assumptions: (1) the instrumental variable is statistically robustly associated with the exposure (e.g., pre-clinical Alzheimer’s disease), (2) there is no confounding between the instrumental variable and outcome (e.g., blood pressure), and (3) the instrumental variable only affects the outcome through its effect on the exposure. 

**Fig. 1:**
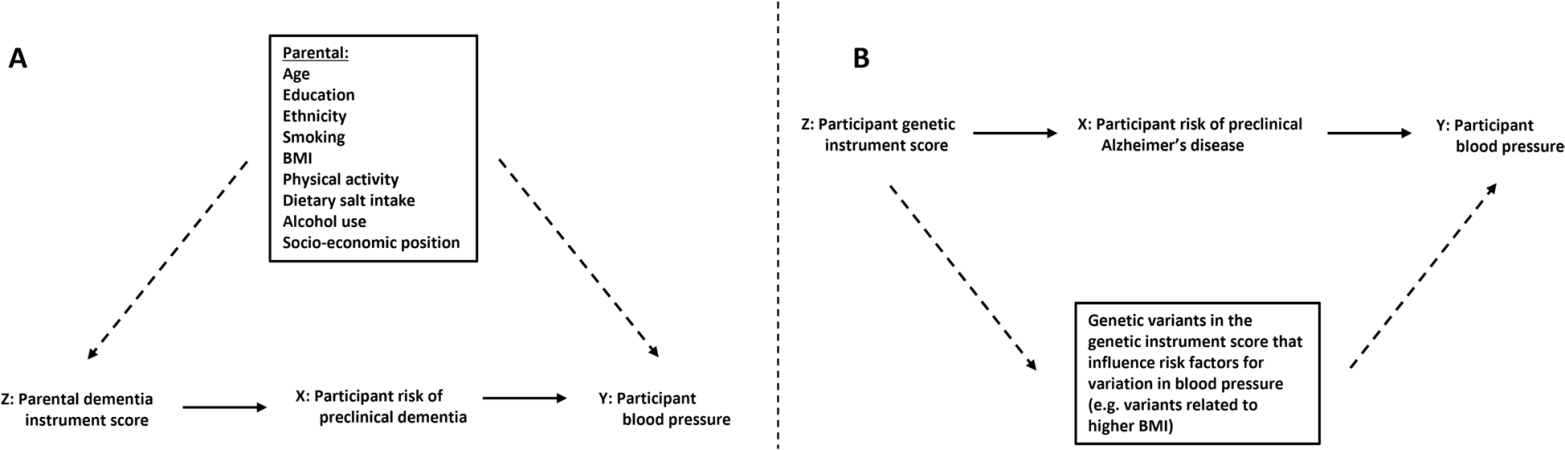
Directed acyclic graphs depicting the hypothesised causal pathways using **A** parental dementia instrument score as an instrumental variable and **B** participant genetic instrument score as an instrumental variable for risk of pre-clinical Alzheimer’s disease. Directed acyclic graphs (DAGs) place arrows between a variable at the base of the arrow and one at the head (point of the arrow) when there is a known or plausible causal effect of the variable at the base on the variable at the top. DAGs are useful for illustrating analysis assumptions and here we use them to show that our two instrumental variables have different key sources of bias)

Table 1 and Fig. 1 illustrate these core assumptions [18]. Instrumental variable analyses can be particularly useful when it is difficult or not possible to measure the exposure, as is the case with pre-clinical Alzheimer’s disease.

**Table 1:** The assumptions underpinning instrumental variable analyses, and how they have been met or violated in this study

*Instrumental variable (IV) assumption*	*How assumption is met or violated*	
	**with PDIS as instrument**	**with PGIS as instrument**
IV1. IV is statistically strongly associated with the exposure of interest in the relevant population	Having one or both parent(s) with clinical dementia is associated with increased risk of developing Alzheimer’s disease themselves	Genetic variants associated at genome-wide significance (*p* < 0.05 × 10^−8^) to AD in a chosen pre-existing AD GWAS[40] were used
IV2. There is no confounding between the IV and outcome	This is likely to be violated by a confounding path between parental and participant education, ethnicity, smoking, physical activity, BMI, and alcohol. We adjusted for participant measures of these. Residual confounding due to lack of parental measures and potential imprecise measures, e.g., of self-report smoking, alcohol and physical activity could bias results away from the null	As genetic variation is fixed at conception, common confounders, such as education, ethnicity, and BMI cannot confound the participant genetic instrument score. Confounding can occur where there are subgroups of different populations (e.g., groups from different ancestral backgrounds) who have different allele frequencies of the AD-related genetic variants, and independently of that have different BP distributions. We limited our analyses to self-declared white European ethnicity to mitigate against that and adjusted genetic analyses for ancestral principal components
IV3. The IV does not influence the outcome by any other path than via the exposure of interest	If parental dementia affected offspring adult blood pressure via a direct path, in addition to a possible effect mediated by offspring pre-clinical dementia this assumption would be violated. We cannot think of such a path and so assume this is not violated	A pathway which can lead to an association between the genetic variants and blood pressure is through horizontal pleiotropy (Fig. 1B). This will be explored with sensitivity analysis, and steps will be taken to minimise
***Additional two-sample MR assumptions***		
MR1. The genetic IV-exposure association study sample and the genetic-outcome association sample are from the same underlying population and have little or no overlap		Both samples were of White European ancestry only and we selected the largest and most appropriate AD GWAS that did not include UK biobank to ensure the two samples were largely independent (non-overlapping)
MR2. The genetic variants are harmonised (same reference allele) across the two samples		Variants will be harmonised, and harmonisation checked by comparing allele frequencies of the reference and effect alleles

*PDIS* parental dementia instrument score, *PGIS* participant genetic instrument score, *MR* Mendelian randomisation, *AD* Alzheimer’s disease, *GWAS* genome-wise association study

Instrumental variable assumptions are described in Table 1 and summarised in relation to this figure here:

1. Relevance assumption – that there is a statistically robust association between the instrument and exposure in the relevant population; shown here by the solid arrow from the parental dementia instrument score (in A) and from the participant genetic instrument score (in B) to risk of pre-clinical Alzheimer’s disease.
2. Independence assumption – that there is no confounding between the instrumental variable and the outcome; because we believe this is plausibly violated for the parental dementia instrument score, we have used dashed arrows to indicate which factors plausibly violate this assumption. For the participant genetic instrument score (in B) there are no arrows suggesting confounding between the participant genetic instrument score and variation in blood pressure, since it is not plausible for factors such as age, sex, BMI, etc. to influence genetic variation. Furthermore, we have controlled for potential confounding due to ancestral subgroups by restricting analyses to European ancestry and used genetic summary data adjusted for ancestral principal components.
3. Exclusion restriction criteria – that the association of the instrument with the outcome is only due to the relation of the instrument with the exposure (i.e. there are no direct paths from the instrument to the outcome that are independent of the exposure. Because we believe it is plausible that genetic variants related to Alzheimer’s disease could relate to other risk factors for variation in blood pressure, we have dashed arrows to show a plausible path from the participant genetic instrument score to participant BP that is independent of the genetic score- Alzheimer’s disease relation (in B), which are not present in the parental dementia instrument score graph (A). Conventional DAG notation is used: Z: instrumental variable; X: exposure being instrumented; Y: Outcome."

Mendelian randomisation is a method that uses genetic variants as instrumental variables to determine the unconfounded effect of potential risk factors on outcomes [19], and is the most common instrumental variable approach used in health research. Mendelian randomisation studies to date have explored the potential effect of higher BP on Alzheimer’s disease and have reported differing results, including evidence for higher BP resulting in lower risk of Alzheimer’s disease, higher risk, and no evidence of effect [20–28]. These differences may be explained by between-study differences in instrument strength, and in the extent to which they explored survival bias and bias due to horizontal pleiotropy. We did not identify any specific Mendelian randomisation study of the effect of Alzheimer’s disease on blood pressure. However, a Mendelian randomisation phenome-wide association study exploring the potential effect of Alzheimer’s disease on various health outcomes suggested that genetic liability to Alzheimer’s disease had an age-related effect on SBP, potentially resulting in higher SBP in younger participants (aged 39–53) but not older participants (53–72 years) [29]. No sensitivity analyses were undertaken for this potential effect.

One hypothesis that could explain some of these inconsistencies is that during the pre-clinical period of Alzheimer’s disease, reduced cerebral blood flow can cause an elevation in peripheral BP in order to protect healthy cerebral perfusion (analogous to the development of renovascular hypertension or pulmonary hypertension in people with increased renal or pulmonary vascular resistance) which benefits cognitive function [30]. However, prolonged elevation of BP over many years leads to structural vascular changes including arteriosclerosis, causing secondary hypoperfusion and blood–brain barrier breakdown, which accelerate the progression of cognitive decline [15]. Thus, it has been hypothesised that there might be a bi-directional relationship between high BP and Alzheimer’s disease [30].

Evidence for the bi-directional Alzheimer’s disease-BP hypothesis has mainly come from biochemical measurements in post-mortem brain tissue [31], as summarised by Love and Miners [30]. Evidence for this hypothesis in cohort studies is limited, with most exploring the association of variation in BP on Alzheimer’s disease. The extent to which these reflect reverse causality, and hence the effect of Alzheimer’s disease on variation in blood pressure, particularly when cohorts span a wide age range and follow-up period, makes the results difficult to interpret. Furthermore, the difficulty of actually measuring pre-clinical Alzheimer’s disease makes it impossible to explore its potential effect on BP in human cohorts. As expected, we did not identify any human cohort studies exploring the effects of pre-clinical Alzheimer’s disease on BP.

The aim of this study was to test the bi-directional Alzheimer’s disease-BP hypothesis in the absence of good measures of cerebral blood flow and/or pre-clinical Alzheimer’s disease in large human epidemiological studies. As the prolonged elevated BP-to-Alzheimer’s disease direction is reasonably well studied, we aim to test the pre-clinical Alzheimer’s disease-to-BP direction of this hypothesis. We address this aim by determining the association of two different instrumental variables for pre-clinical Alzheimer’s disease with subsequent BP, in humans not, or not yet, experiencing prodromal or clinical Alzheimer’s disease. The first instrument was a “parental dementia instrument score,” with participants given a score based on their self-report of whether neither one nor two of their parents had been diagnosed with all-cause dementia, adjusted for parental age to reflect our confidence in likelihood of dementia diagnosis (i.e., younger parents may have gone on to develop dementia had data been collected later or had they lived longer). The second instrument approach was Mendelian randomisation, using a “participant genetic instrument score,” as the instrumental variable. This was constructed by generating a weighted allele score of alleles in single-nuclear polymorphism (SNPs) that in a large genome-wide association study (GWAS) are significantly associated with odds of Alzheimer’s disease.

The value of using these two different instrumental variables is that they have different and unrelated key sources of bias, so if both are associated with higher BP in the same direction that would provide stronger evidence for the hypothesis than if we had results from just one of them [32]. The key potential source of bias in the parental dementia instrument score is confounding between that instrument and BP, whereas the key potential bias in the participant genetic instrument score is unbalanced horizontal pleiotropy resulting in a direct path from the instrument to the outcome, independent of Alzheimer’s disease (Table 1 and Fig. 1).

## Methods

### Study population and analyses samples

We used data from the UK Biobank, a large prospective cohort study that recruited 503,325 adults (5.5% of those invited) aged between 40 and 69 years and recruited from across the UK between 2006 and 2010 [33]. Of the 503,325, 912 were excluded because they had withdrawn their data from the study. We published all methods of data preparation, validation, and analysis online a priori prior to running analyses [34] and this study followed the Strengthening the Reporting of Observational Studies in Epidemiology (STROBE) reporting guidelines [35] (Additional file 1: Table S1).

We excluded 993 participants who had been diagnosed with prevalent dementia, or incident dementia within the first 5 years of recruitment, to avoid reverse causality. Prevalent dementia (*n* = 89) was obtained from the baseline self-completed diseases questionnaire. Information on incident cases (*n* = 904) was obtained from available linked primary care records and hospital episode statistics (see Additional file 1: Table S2 for ICD-9 and ICD-10 codes used for exclusion). Of the remaining 501,420 participants, 445,911 had a valid parental dementia instrument score, and 336,946 had a valid participant genetic instrument score (excluding those that failed quality control analyses run using a previously developed QC pipeline [36], as well as non-European participants to avoid potential confounding by population stratification). Missing outcome and confounder data were less than 0.5%, other than education which was missing for 1.5% participants (Table 2 and Additional file 1: Table S3).

**Table 2:** Description of participants included in each analysis

**Observed variable**	**All eligible participants (PDIS cohort)**		Complete data, PDIS analysis cohort**		Complete data, PGIS analysis cohort***	
***Categorical***	*n*(%)	N*	*n*(%)	*N*	*n*(%)	*n*
Women	272,930 (54)	501,420	239,901 (55)	433,764	180,776 (54)	336,295
Education, highest qualification CSE/O-level/GCSE or equiv	217,047 (43)	492,187	185,224 (43)	433,764	148,852 (44)	333,212
Ethnic minority groups	26,968 (5)	498,659	20,254 (5)	433,764	0 (0)	NA
Current smoker	52,845 (10)	498,500	43,238 (10)	433,764	33,276 (10)	334,827
High salt diet (always add salt to food)	24,363 (5)	500,317	19,931 (5)	433,764	14,420 (4.3)	335,969
Use alcohol daily/almost daily	101,567 (20)	499,944	89,742 (21)	433,764	72,029 (21)	335,772
Do 10 min or more of vigorous physical activity 3 or more times per week	181,982 (36)	501,420	153,852 (36)	433,764	118,739 (35)	336,005
***Continuous***	***mean (sd)***	*N*	***mean (sd)***	*N*	***mean (sd)***	*N*
Age in years	56.5 (8.1)	501,420	56.4 (8.0)	433,764	56.9 (8.0)	336,295
SES by TDI	− 1.3 (3.1)	500,798	− 1.4 (3.0)	433,764	− 1.6 (2.9)	336,005
BMI	27.4 (4.8)	498,673	27.3 (4.8)	433,764	27.4 (4.8)	335,339

*PDIS* parental dementia instrument score, *PGIS* participant genetic instrument score, *SES* socioeconomic status, *TDI* Townsend deprivation index, *SBP* systolic blood pressure, SD standard deviation

^*^With available data for that variable

^**^Complete data for PDIS-SBP associations and fully adjusted model

^***^Complete data for PGIS-SBP associations and additional exclusions

### Exposure instrumental variables

We used parental diagnosis of all-cause dementia as one of our instrumental variables for risk of Alzheimer’s disease in offspring. Information on parental all-cause dementia was obtained from the baseline self-completed electronic questionnaire. Participants were asked to report whether their mother or father had ever suffered from Alzheimer’s disease or dementia, and to report each parent’s current age if still alive, or age at death if not. From this information we created a weighted instrumental variable for risk of pre-clinical dementia with an approach similar to that used in a previous study [37]. This weighting means that each parent diagnosed with dementia contributed one full unit to the participant’s instrument score, whilst each parent without dementia contributed less than one unit, inversely proportional to their age, reflecting our confidence in their not having dementia, such that weight = (100 − age)/100 (age 100 is approximately the 95th percentile for life expectancy in developed countries). We used a cap at age 68 so that any parent aged 68 years or younger at the point of information collection, without diagnosed dementia, contributed the same level of (low) weight (because dementia diagnosis rises steeply with increasing age above age 68, but remains very low under age 68) [38, 39]. For example, if a parent reached 100 without a dementia diagnosis, we are confident in giving 0 weight to the instrumental variable score for pre-clinical dementia. However, if a parent was 68 or younger without a dementia diagnosis, we could not be confident in giving 0 weight to the instrumental variable score for pre-clinical dementia, so gave a higher contribution of 0.32. We refer to this instrumental variable score as “parental dementia instrument score.”

We also used genetic liability to Alzheimer’s disease (SNPs) as an instrumental variable for risk of pre-clinical Alzheimer’s disease. This instrumental variable was generated by creating a weighted participant genetic instrument score of genome-wide significant (*p* ≤ 0.05 × 10^−8^) SNPs selected from a previously published GWAS [40] and weighted by the magnitude of the association with Alzheimer’s disease from that GWAS. We chose this Alzheimer’s disease GWAS for the following reasons: (1) It has close case–control matching by mean age of assessment (72.9 years for cases and 72.4 years for controls); (2) 100% of cases and 86.2% of controls had undergone clinical or pathological assessment; and (3) it did not contain data from UK Biobank, which we use for our main analyses. We used a conservative pruning threshold to keep weakly correlated SNPs and non-correlated SNPS: Correlated SNPs were pruned using the European subsample of the 1000 genomes project, with R^2^ < 0.01 and a window of < 10,000 kb. Where correlated above this threshold we selected the SNP with the strongest Alzheimer’s disease association to remain in the score, which led to the selection of 32 SNPs that were associated with Alzheimer’s disease (Additional file 1: Table S4). We calculated a weighted score for pre-clinical Alzheimer’s disease by multiplying the number of effect alleles for each participant in UK Biobank by the weight (association of the SNP with Alzheimer’s disease from the GWAS), then summing across all 32 SNPs. We refer to this instrumental variable score as “participant genetic instrument score.”

### Relevance of the instrumental variables

It is not possible for us to determine instrument strength for either of our instrumental variables, as we do not have a measure of pre-clinical Alzheimer’s disease, indeed as noted above the near impossibility of measuring pre-clinical disease is why the instrumental variable approach provides a means to address our hypothesis. We assessed the association of each instrument with diagnosed clinical dementia, to explore the relevance of the instrument (i.e., if our instruments are relevant, they should associate with higher risk of diagnosed dementia).

### Outcomes

Two BP measurements were taken by trained staff on each participant, usually using an automated device (Omron Digital BP monitor), as part of the in-person assessment. We used the mean of both automated measurements (or manual readings if automated readings were missing) of systolic BP (SBP) from the baseline assessment to generate a continuous SBP variable. If only one reading was recorded at baseline assessment, we took this as the mean SBP. If both readings were missing at baseline assessment, we used the mean of both readings at a subsequent visit instead. We used the same procedure to generate the continuous diastolic BP (DBP) variable.

We generated a binary outcome variable of hypertension, by combining those who self-reported essential hypertension, with those taking an anti-hypertensive medication (detailed below), as well as those with a SBP measurement above 140 mmHg or those with a DBP measurement above 90 mmHg. This identified those with unknown, undiagnosed hypertension, as well as those with known hypertension. A Venn diagram showing the overlap between self-report hypertension, taking anti-hypertensive medications, high SBP, and high DBP is provided in Additional file 1: Fig. S1.

For any participants who were on antihypertensive medication, or on medications that were prescribed for other conditions, such as heart failure, but that also reduce BP, we applied + 15 mmHg to SBP and + 10 mmHg to DBP, which are conservative estimates of average BP lowering by antihypertensive medications [41]. Information on use of medications was obtained at the baseline assessment. Participants were asked to bring along all packets of prescribed medication they usually took (not purchased over-the-counter and not prescribed as a one-off) and these were recorded by nurses.

### Confounders and covariables

The parental dementia instrument score could violate the assumption of no confounding between the instrumental variable and outcome, whereas confounding is not plausible for the participant genetic instrument score (Fig. 1 and Table 1). We considered the following to be confounders for the parental dementia instrument score analyses based on their known or plausible influence on Alzheimer’s disease and BP: age, sex, education, ethnicity, smoking, BMI, physical activity, dietary salt intake, alcohol use, and socio-economic position. Ideally, we would have had data on parental and participant measures of these. We only had measures on the participants and demonstrate in Fig. 1 why we consider controlling for participant confounders should block the confounding path. Details of how these variables were assessed are reported in Additional file 1: Supplementary Methods.

### Statistical analyses

We summarised baseline characteristics for all eligible participants using data from both analysis cohorts and presented as percentages for categorical variables and means with standard deviations (SDs) for normally distributed continuous variables.

We used multivariable linear regression to determine the association of each instrument score with SBP and DBP and logistic regression for their associations with hypertension. With the parental dementia instrument score, we added each confounder one-by-one and assessed the standard error for evidence of multicollinearity or missing data effects on standard error. We then corrected BP for anti-hypertensive medication use. Our a priori assumption was that for the parental dementia instrument score, our final model would be the full confounder-adjusted model with BP correction. With the participant genetic instrument score as instrument, our a priori assumption was that genetic variants would not be associated with potentially confounding factors (but we checked this using linear or logistic regression). However, as the GWAS estimates used in our participant genetic instrument score had been adjusted for age and sex, for consistency our main model was adjusted for genetic principal components, age, and sex, with BP correction for anti-hypertensive medication use.

### Sensitivity analyses

For the participant genetic instrument score, we performed leave-one-out analyses of SNPs in the participant genetic instrument score to assess any influential outliers.

We ran analyses that included additional variables that may be potential confounders of the association between the parental dementia instrument score and each outcome: continuously measured glycated haemoglobin (HbA1c, a measure that reflects glucose levels over ~ 4 months), total cholesterol, high-density lipoprotein cholesterol (HDLc), low-density lipoprotein cholesterol (LDLc), triglycerides, and c-reactive protein (CRP), to see if addition of these variables altered our effect estimates.

## Results

### Descriptive analyses

The distributions of all potential confounding factors were very similar in the analysis cohort and in the complete data cohort for both the parental dementia instrument score and participant genetic instrument score (Table 2). All eligible participants had mean age 56.5, SD 8.1 years, with 54% female, had a mean Townsend deprivation index (TDI) score − 1.3, SD 3.1 and mean BMI 27.4, SD 4.8 kg/m^2^. Participants contributing to parental dementia instrument score analyses had mean age 56.4, SD 8.0 years, with 55% female, had a mean TDI score − 1.4, SD 3.0 and mean BMI 27.3, SD 4.8 kg/m^2^. This compares similarly to the participant genetic instrument score analyses which had mean age 56.9, SD 8.0 years, with 54% female, mean TDI score − 1.6, SD 2.9 and mean BMI 27.4, SD 4.8 kg/m^2^. The only difference of note is that the parental dementia instrument score analyses was made up of 5% ethnic minority groups, but the participant genetic instrument score analyses were restricted to White Europeans.

### Instrumental variable associations with blood pressure

**Fig. 2:**
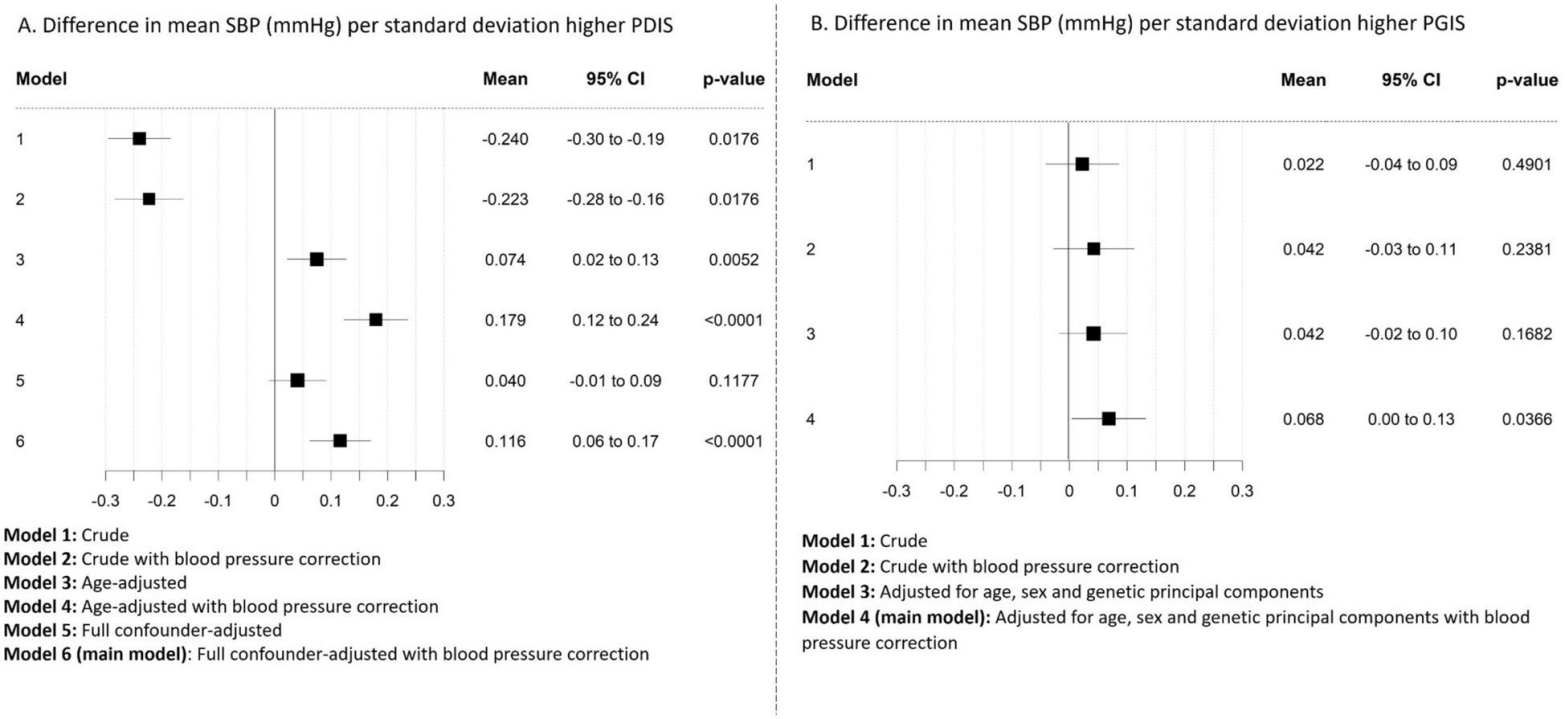
Difference in mean systolic blood pressure (SBP, mmHg) (black squares), and 95% confidence intervals (horizontal lines) per one standard deviation higher parental dementia instrument score (PDIS, **A**) as instrumental variable for risk of pre-clinical Alzheimer’s disease and participant genetic instrument score (PGIS, **B**) as instrumental variable for risk of pre-clinical Alzheimer’s disease. Each model (from crude to fully adjusted) is shown to indicate the differing effects of confounding with each instrument (due to each being vulnerable to differing biases (see main text for details))

**Fig. 3:**
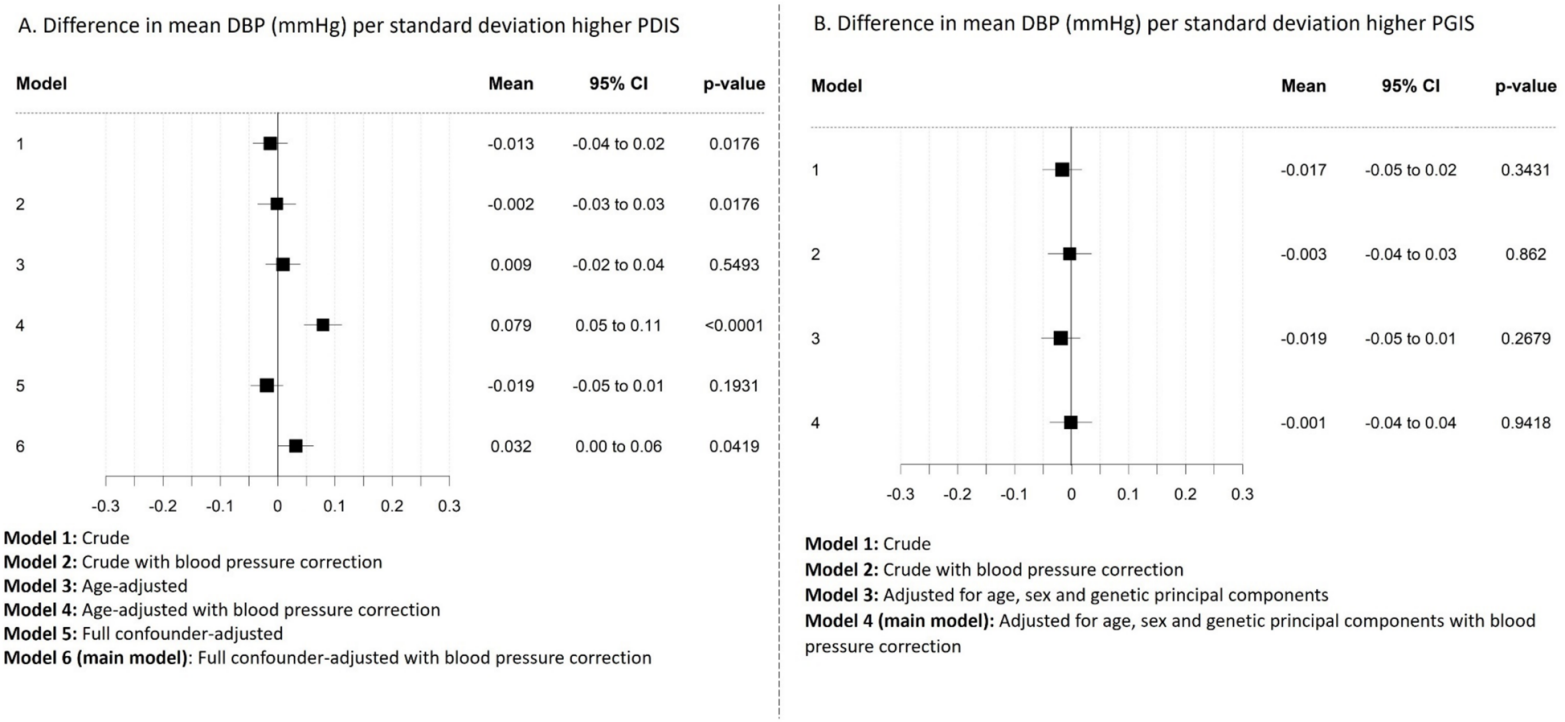
Difference in mean diastolic blood pressure (DBP, mmHg) (black squares), and 95% confidence intervals (horizontal lines) per one standard deviation higher parental dementia instrument score (PDIS, **A**) as instrumental variable for risk of pre-clinical Alzheimer’s disease and participant genetic instrument score (PGIS, **B**) as instrumental variable for risk of pre-clinical Alzheimer’s disease. Each model (from crude to fully adjusted) is shown to indicate the differing effects of confounding with each instrument (due to each being vulnerable to differing biases (see main text for details))

**Fig. 4:**
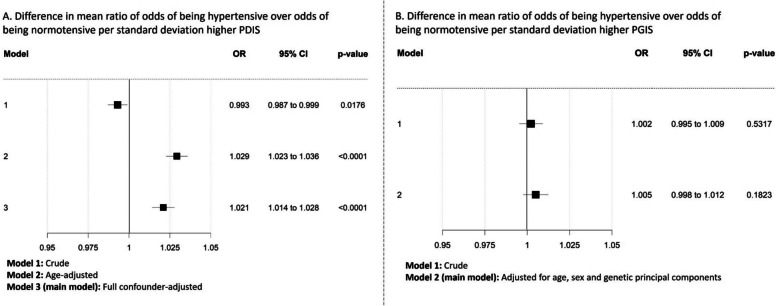
Difference in mean ratio of odds of being hypertensive over odds of being normotensive (odds ratio, black squares), and 95% confidence intervals (horizontal lines) per one standard deviation higher parental dementia instrument score (PDIS, **A**) as instrumental variable for risk of pre-clinical Alzheimer’s disease and participant genetic instrument score (PGIS, **B**) as instrumental variable for risk of pre-clinical Alzheimer’s disease. Each model (from crude to fully adjusted) is shown to indicate the differing effects of confounding with each instrument (due to each being vulnerable to differing biases (see main text for details))

Using the analysis samples for each instrument, and focusing primarily on the fully adjusted models, we found evidence to suggest that higher risk of pre-clinical Alzheimer’s disease, as instrumented both by the parental dementia instrument score (+ 0.116 mmHg (95% confidence interval (CI) 0.06 to 0.17) SBP per one SD higher PDIS, *p* < 0.0001) and the participant genetic instrument score (+ 0.068 mmHg (95% CI 0.00 to 0.13) SBP per one SD higher PGIS, *p* = 0.037), carried a liability to higher mean SBP (Fig. 2A and B respectively). We found no strong evidence to suggest that higher risk of pre-clinical Alzheimer’s disease, as instrumented by either score, carried a liability to higher mean DBP (+ 0.032 mmHg (95% CI 0.00 to 0.06) DBP per one SD higher PDIS, *p* = 0.042; − 0.001 mmHg (95% CI − 0.04 to 0.04) DBP per one SD higher PGIS, *p* = 0.942; Fig. 3A and B respectively). Higher risk of pre-clinical Alzheimer’s disease, as instrumented by the parental dementia instrument score, was associated with higher odds of being hypertensive (odds ratio(OR) 1.021 (95% CI 1.014 to 1.028) per one SD higher PDIS, *p* < 0.0001; Fig. 4A), with a weaker positive association when instrumented by the participant genetic instrument score (OR 1.005 (95% CI 0.998 to 1.012) per one SD higher PGIS, *p* = 0.182; Fig. 4B).

### Relevance of the instrumental variables

Both instruments were positively associated with diagnosed dementia in the participants (OR 3.23, 95% CI 2.60 to 4.01, *p* < 0.0001 for the parental dementia instrument score and OR 1.62, 95% CI 1.54 to 1.70, *p* < 0.0001 for the participant genetic instrument score; Additional file 1: Table S5).

### Effect of age and other potential confounders

With the parental dementia instrument score analyses, the association between risk of pre-clinical Alzheimer’s disease and SBP changed from a negative association (reduction in SBP (difference in mean − 0.22 mmHg (95% CI − 0.28 to − 0.16), *p* = 0.018) per one SD higher parental dementia instrument score) for the unadjusted corrected analysis; Fig. 2A, Model 2) to a positive association (an increase in SBP (mean + 0.18 mmHg (95% CI 0.12 to 0.24), *p* < 0.0001) per one SD higher parental dementia instrument score) after adjustment for participant age at BP measurement (Fig. 2A, Model 4). A similar change in direction of association was also observed for DBP (mean − 0.002 mmHg (95% CI − 0.03 to 0.03), *p* = 0.018 for unadjusted corrected analysis; Fig. 3A, Model 2, to mean + 0.08 mmHg (95% CI 0.05 to 0.11), *p* < 0.0001; Fig. 3A, Model 4, after for adjustment for age) and hypertension (OR 0.993 (95% CI 0.987 to 0.999), *p* = 0.018; Fig. 4A, Model 1 to OR 1.029 (95% CI 1.023 to 1.036), *p* < 0.0001; Fig. 4A, Model 2, after adjustment for age).

In contrast, as expected for a genetic score, with the participant genetic instrument score analyses, point estimates for SBP, DBP, or hypertension did not change substantially with adjustment for age, sex, or principal components (+ 0.042 (95% CI − 0.03 to 0.11), *p* = 0.238; Fig. 2B, Model 2 and + 0.068 (95% CI 0.00 to 0.13), *p* = 0.0366; Fig. 2B, Model 4 for SBP; − 0.003 (95% CI − 0.04 to 0.03), *p* = 0.862; Fig. 3B, Model 2 and − 0.001 (95% CI − 0.04 to 0.04), *p* = 0.942; Fig. 3B, Model 4 for DBP and OR 1.002 (95% CI 0.995 to 1.009), *p* = 0.532; Fig. 4B, Model 1 and OR 1.005 (95% CI 0.998 to 1.012), *p* = 0.1823; Fig. 4B, Model 2 for hypertension). Results from all models are also available in Additional file 1: Tables S6-S8.

### Sensitivity analyses

In the leave-one-out sensitivity analyses for the participant genetic instrument score, removal of each SNP one at a time produced results that were consistent with the result that included all SNPs suggesting that there were no individual SNPs in the participant genetic instrument score that substantially influenced results (Additional file 1: Figs. S2-S4). The addition of potential confounding by HbA1c, total cholesterol, HDLcc, LDLc, triglycerides, and CRP in the parental dementia instrument score model with each outcome showed consistent results to our main model on participants with complete data for these additional variables (+ 0.089, *p* = 0.003 to + 0.099, *p* = 0.001 for SBP, Additional file 1: Table S9; + 0.014, *p* = 0.402 to + 0.015, *p* = 0.381 for DBP, Additional file 1: Table S10; and OR = 1.018, *p* < 0.0001 to OR = 1.019, *p* < 0.0001 for hypertension, Additional file 1: Table-S11). When we restricted the main model to those with complete data on all confounders (main and additional) there was some evidence of selection bias, with associations in this complete case subsample being closer to null compared to the larger sample (SBP mean from + 0.116 to + 0.089 mmHg per one SD higher parental dementia instrument score; see Additional file 1: Table S9; DBP mean from + 0.032 to + 0.014 mmHg per one SD higher parental dementia instrument score; see Additional file 1: Table S10; and hypertension OR from 1.021 to 1.018; see Additional file 1: Table S11).

## Discussion

In this large human study, we have found preliminary evidence that SBP (but not DBP) is elevated in people at risk of pre-clinical Alzheimer’s disease but without any clinical evidence of cognitive impairment or dementia. To the best of our knowledge, this is the first cohort study to test the pre-clinical Alzheimer’s disease-to-BP direction of the bi-directional Alzheimer’s-BP hypothesis by providing good evidence that higher risk of pre-clinical Alzheimer’s disease leads to higher SBP and hypertension.

There is evidence from observational studies for the other direction (BP-to Alzheimer’s disease) of the bi-directional Alzheimer’s-BP hypothesis, that mid-life (but not late-life) hypertension is associated with increased risk of developing Alzheimer’s disease [8–13]. Two RCTs found that treatment of hypertension had no effect on Alzheimer’s disease risk, which could suggest that the results from previous observational studies is due to confounding; however, one trial did not have a suitable control group for these purposes [16], and the other trial was underpowered due to being terminated early [17], so this is not necessarily the case.

We used two complementary instrumental variables of a risk of pre-clinical Alzheimer’s disease to enable the assessment of pre-clinical dementia which is difficult to directly measure. These complement each other by having different key sources of bias, which was a key rationale for us using these two instruments as we could infer causality when both results were consistent. The key source of bias for the parental dementia instrument score is confounding due to age, smoking, body mass index, and other factors that would cause parental dementia and variation in offspring BP. By contrast the genetic instrument score is less likely to be influenced by such confounders as genetic variation is fixed at conception and cannot be changed by someone’s age, smoking, body mass, or similar factors. The change in direction of associations for the parental dementia instrumental score with adjustment for age, with no change in the genetic instrument score with age, sex, or principal component adjustment, supports the importance of confounding with the parental dementia instrument score, particularly by age. By taking parental age into account in our parental instrument score, it is less prone to survival bias than a simple “count” score of whether a parent had been diagnosed with dementia. If using a simple count, the accuracy of the score is associated with parental age (the older the parents the more accurate the score), as for a dementia diagnosis they will have had to reach that age. As BP is also heritable, and increases mortality risk, we would expect an inverse association between BP and a simple parental count score, largely driven by survival bias due to differential misclassification of non-dementia (that would have been scored as parental dementia had parents lived longer or participants been assessed later). Our parental instrument score accounts for age, to represent our certainty of correct classification of non-dementia, and thus is less prone to, although still not possible to be free from, survivor bias. Our participant genetic instrument score analyses should not be impacted by confounding, but the key source of bias for these analyses is horizontal pleiotropy. These key sources of bias are discussed more in the limitations section below, but the consistency of direction of association across the two instruments, with higher mean SBP and higher odds of hypertension demonstrated in both, despite their differences in biases, supports the hypothesis that a risk of pre-clinical Alzheimer’s disease causes a rise in SBP.

Our results have relevance to previous studies suggesting possible complex relations between BP and Alzheimer’s disease, and address many of the limitations of those previous studies. The large sample size enabled us to exclude participants with prevalent and incident dementia in the first 5 years of follow-up, so that associations reflect pre-clinical disease and importantly are unlikely to be driven by established dementia or cognitive impairment as a consequence of prolonged elevation of BP. The only other cohort study that we know of that has explored the effect of Alzheimer’s disease on BP did not exclude these participants but did find that genetic liability to Alzheimer’s disease resulted in higher SBP in younger participants (aged 39–53) [29], most of whom would have therefore been at a pre-clinical stage of pathology (of those who would go on to develop Alzheimer’s disease).

### Mechanisms: why might pre-clinical Alzheimer’s disease cause higher SBP?

We found that risk of pre-clinical Alzheimer’s disease, as measured using both a parental dementia instrument score and a participant genetic instrument score, associates with higher peripheral SBP (but not DBP). We hypothesise that this is due to very early reductions in cerebral perfusion. This is consistent with the physiological and laboratory studies informing each part of the full hypothesis: cerebral blood flow is reduced early in Alzheimer’s disease [2, 3], for example, decreases in grey-matter cerebral blood flow were documented in young individuals (aged 19–32) at risk of familial Alzheimer’s disease [42]; reduced cerebral blood flow can increase SBP [30]; and cerebral infusion of beta-amyloid (an Alzheimer’s disease pathological hallmark) in rats caused a progressive rise in peripheral BP that was associated with increased levels of the vasoconstrictor endothelin-1 within the brain [43, 44]. Cerebral hypoperfusion is a central and proximal process in the pathophysiology of early Alzheimer’s disease, rather than simply a consequence of diminished metabolic demand. Reversing the reduction (and misdirection) of cerebral blood flow could restore cognitive function, provided synaptic and neuronal damage is not too advanced [45]. Recognising physiological effects of reduced cerebral blood flow, such as elevation of peripheral BP in some people, could play an important part in screening for cerebral vascular dysfunction and identifying those in whom therapeutic interventions might be tested. As more detailed scanning data are collected on UK Biobank participants and as longer-follow up into older age is undertaken, in coming years it will be possible to explore the associations of our instruments for risk of pre-clinical Alzheimer’s disease with cerebral blood flow, and of both of these with subsequent incident Alzheimer’s disease.

We did not find evidence that pre-clinical Alzheimer’s disease affected DBP when using either the parental dementia instrument score or the participant genetic instrument score. Some studies have suggested a similar DBP-Alzheimer’s disease relationship to SBP, in that elevated mid-life DBP and low late-life DBP is associated with Alzheimer’s disease [46]. However, DBP has also shown a complex relationship with age where DBP peaks at age 50 due to increased systemic vascular resistance and then declines thereafter [47]. There are multiple determinants of DBP, making it complex: The key factors are vasoconstrictor tone of arterioles (smaller radius usually means a higher DBP) and stiffness of the arteries. The arteries store the energy needed for DBP so that more compliant vessels usually mean a higher DBP [48]. The compliance of an artery is related to both the pressure within the vessel (i.e., the pressure during systole; higher pressures can reduce compliance) [49] and basal tone of the vessel (where decreased radius due to smooth muscle contraction decreases compliance and increases stiffness). Therefore, in a less compliant/stiffer artery which has a higher SBP; this can reduce the DBP. This scenario is often seen in ageing where DBP decreases but SBP increases [47]. Taken together, this could mean that DBP is not a good predictor of dementia-related outcomes because lower DBPs are sometimes a result of stiffer less compliant vessels. In line with this, the Framingham Heart study found that SBP was a better predictor of cardiovascular outcomes vs. DBP [50].

### Study limitations

#### Design

As noted above we are unable to test the hypothesis fully due to the lack of information on cerebral blood flow. Ideally, we would want a large human study with repeat measures of cerebral blood flow and BP such that the bidirectional hypothesis could be tested further with our instrumental variables. Currently, no such study exists, though the global increase in very large human resources like UK Biobank that increasingly have repeat imaging data, and further follow-up of participants, could make this possible in the future.

We used available linked primary care and hospital admission data to exclude participants with diagnosed prevalent and 5-year follow-up incident diagnosed Alzheimer’s disease to avoid reverse causality. Linked primary care data were only available on 45% of the cohort when we conducted our analyses, and that remains the case currently (linked hospital data are available on all participants). Thus, it is possible we failed to include some participants with Alzheimer’s disease who had the diagnosis only recorded in primary care and who were amongst the 55% not linked to primary care data. Analysis of the subset that have both hospital data and primary care data showed that the age-specific cumulative incidence of dementia is more than halved when primary care data are not included [51], as dementia is primarily diagnosed and managed in primary care. This misclassification could have biased our main results by a failure to exclude patients with cognitive impairment or clinical Alzheimer’s disease. We chose a 5-year follow-up a priori although recognise that it could be beneficial for future studies to exclude patients who develop dementia over an even longer follow-up period, in addition to obtaining primary care record linkage for the whole cohort, to be able to better isolate pre-clinical disease processes.

Whilst instrumental variables can provide a solution to impossible or difficult to measure exposures, this is not the case for outcomes. In the absence of suitable datasets that include information on preclinical Alzheimer’s disease (e.g., by detection of related biochemical changes), instrumental variable analyses, including Mendelian randomisation analyses to test whether BP influences pre-clinical Alzheimer’s disease are not currently possible.

#### Strength and validity of instrumental variables

The inability to measure pre-clinical Alzheimer’s disease means we cannot test the instrument strength for either instrumental variable. We did find a positive association of both instrumental variables with diagnosed all-case dementia, providing some evidence of the relevance of our instruments. The expected bias in any instrumental variable analysis undertaken in a single sample, as here, is that the bias would be towards the confounded association. For the genetic score instrument this would be mitigated to some extent by weighting the score by association magnitudes from an independent GWAS. For the parental score instrument, we expected there to be confounding and selected a key set of confounders to adjust for a priori. That we did not see any change with the addition of other potential confounders is reassuring.

In the parental dementia instrument score, we were not able to focus specifically on Alzheimer’s disease as the baseline questionnaire only collected whether the participant’s mother or father had ever suffered from Alzheimer’s disease or dementia. Although Alzheimer’s disease is the most common form of dementia, it accounts for 60–80% of all dementia cases [52], which means 20–40% of parental cases that contributed to the score did not have Alzheimer’s disease, which could lead to weak instrument bias. We included biological parents and those who had adopted the UK biobank participant. We did this because the parental dementia instrument should capture environmental factors as well as genetic factors. Of the total 501,420 included in the main analyses, 7414 (1.48%) had adopted parents, meaning these are unlikely to have had any major impact on the results.

In the participant genetic instrument score, the SNPs had been selected for being associated with Alzheimer’s disease, so this score is perhaps a more specific instrument for risk of pre-clinical Alzheimer’s disease, although it should be noted that most of the diagnoses in GWASs used to establish the participant genetic instrument score were of clinically probable rather than the “gold standard” neuropathologically confirmed Alzheimer’s disease which requires post-mortem examination. However, the positive predictive value of a diagnosis of probable Alzheimer’s disease, made using the criteria used for those GWASs, is > 80% [53], and the fact that the results are in a consistent direction for both instruments despite differences in relevance/specificity is reassuring.

#### Potential biases and how they were addressed

The assumptions underpinning the validity of our analyses are set out in detail in Table 1 and Fig. 1. The key source of bias for our analyses with the parental dementia instrument score is likely to be confounding. We adjusted for participant measures of confounders we felt were plausible and were decided a priori in our analysis plan (published before we started analyses), but there may be more confounding factors that we did not include. There may also be residual confounding due to lack of parental measures of these factors, as well as the potential for imprecise measures, for example of self-report smoking, alcohol, and physical activity could bias our results away from the null. As a sensitivity analyses, we included additional variables to our main model for each outcome, that might associate with dementia and blood pressure, but whether they are causal is less clear. This showed consistent results to our main model, with perhaps the addition of some selection bias (see Additional file 1: Tables S9-11). There is a potential issue that parents being diagnosed with dementia might influence offspring behaviours that control glucose and lipids, leading to these factors being potential mediators. These consistent sensitivity tests lend confidence to our results.

For our analyses with the participant genetic instrument score, metabolic and behavioural factors, or pre-existing disease etc. cannot cause variation in allele frequency (as genetic variation is fixed at conception) and so cannot confound these associations [19, 54]. However, confounding can occur when there are subgroups of different populations (e.g., groups from different ancestral backgrounds) who have different allele frequencies, and independently of that have different BP distributions. To mitigate this, we restricted to self-declared white European ethnicity and adjusted for ancestral principal components, as is standard. Instead, the key source of bias for our analyses with the participant genetic risk score is through horizontal pleiotropy, which we cannot rule out here, though our leave-one-out analyses suggest no single SNP has a major effect on the main result, and the consistency across results after removing each SNP individually provides some support for horizontal pleiotropy not having a major effect. We have not explored unbalanced horizontal pleiotropy using methods such as MR-Egger regression or weighted median as these were developed specifically for two-sample MR. Whilst there has been some development to enable them to be used in one-sample MR [55], they are still not widely used. Sensitivity analyses that adjust for plausible causes of variation in blood pressure did not alter the participant genetic instrument score analyses. Furthermore, the fact that results are consistent between two analyses suggests that neither unbalanced horizontal pleiotropy in the participant genetic instrument score, nor confounding in the parental dementia instrument score, are important drivers of the results.

For the same argument for confounding as discussed above, reverse causality is implausible for the participant genetic instrument score as BP cannot affect allele frequency. Similarly, where cross-generational exposure instrumental variables are used, as for our parental dementia instrument score, reverse causality is not plausible as offspring BP cannot plausibly affect parental dementia [56–58].

## Conclusions

Our detailed analyses using two different instrumental variables provides evidence that SBP, but not DBP, is elevated in people at risk of pre-clinical Alzheimer’s disease but without any clinical evidence of cognitive impairment or dementia. As currently there are no ways to measure pre-clinical Alzheimer’s disease, our results of the association of two instrumental variables with blood pressures are an estimate of the effect of pre-clinical Alzheimer’s disease on BP. They provide evidence that pre-clinical Alzheimer’s disease results in higher blood pressure, and whilst we cannot make inferences about the magnitude of any effect, our results support our prior hypothesis and previous physiological, laboratory, and animal studies informing each part of our full hypothesis, and we hope will stimulate further studies of this hypothesis. Genetic instrumental variable analyses have been previously used to explore risk factors for Alzheimer’s disease, including effects of disrupted sleep, BP, and lipid-lowering drug targets [20–28, 59, 60]. We are also aware of one previous study that compared results from a family-based instrument and a genetic instrument in UK biobank data, as we have (in that case to explore intergenerational BMI effects) [58]. Beyond that study and ours, we are not aware that any other studies that have compared family-based and genetic instrumental variables to address a health question. We have obtained preliminary evidence that pathogenic processes in pre-clinical Alzheimer’s disease increase BP. Obtaining a better understanding of the changing relationship with BP at different stages of Alzheimer’s disease may enable optimisation and targeting of therapies more effectively. Perhaps midlife hypertension should prompt investigation of possible preclinical Alzheimer’s disease, for example, by measurement of phospho-Tau-217, −181, and −231 in blood, with follow-up investigations as indicated [61–63]. Additional research is needed to optimise antihypertensive treatment strategies in relation to age and Alzheimer’s disease biomarkers. A key question is which classes of antihypertensives can maintain cerebral blood flow whilst lowering peripheral BP, yet good evidence from randomised controlled trials is lacking (cerebral blood flow was not measured in existing trials of antihypertensives and follow-up was not long enough to determine dementia status). Our understanding is limited to observational evidence, which suggests treatment with calcium channel blockers or angiotensin II receptor blockers (ARBs) to be associated with lower dementia risk than treatment with other antihypertensives [64]. Other meta-analyses of observational studies have found ARBs but not ACE inhibitors led to significant reduction in risk of dementia [65]. More research is needed to be able to consider the feasibility of personalised BP management strategies for Alzheimer’s disease prevention.

Human population studies can use this design in the future to identify pre-clinical disease processes, increase our understanding of the early pathogenic cascades, and inform strategies to delay or prevent Alzheimer’s disease. 

## Supplementary Information

Additional file 1: Tables S1-S11; Figures S1-S4; Supplementary Methods. Table S1 – STROBE Statement. Table S2 – ICD-9 and ICD-10 codes for dementia. Table S3 – Missing data. Table S4 – SNP information. Table S5 – Association of each instrument with all-cause dementia. Table S6 – SBP results for all models. Table S7 – DBP results for all models. Table S8 – Hypertension results for all models. Table S9 – Sensitivity analyses (SBP). Table S10 – Sensitivity analyses (DBP). Table S11 – Sensitivity analyses (hypertension). Figure S1 – Venn diagram for hypertension variable. Figure S2 – Leave-one-out analyses (SBP). Figure S3 – Leave-one-out analyses (DBP). Figure S4 – Leave-one-out analyses (hypertension). Supplementary Methods – Assessment of confounders

## Data Availability

No datasets were generated or analysed during the current study.

## Abbreviations

BP: Blood pressure
CI: Confidence interval
DBP: Diastolic BP
GWAS: Genome-wide association study
OR: Odds ratio
RCT: Randomised controlled trial
SBP: Systolic BP
SD: Standard deviation
SNPs: Single-nuclear polymorphism
STROBE: Strengthening the reporting of observational studies in epidemiology
