# Supplementary material for: Exploring the effect of pre-clinical Alzheimer’s disease on blood pressure using Mendelian randomisation and parental dementia as an instrumental variable in UK Biobank
Source: BMC Med. 2025 Aug 20;23:483. doi: 10.1186/s12916-025-04295-5 (PMC12366137; doi:10.1186/s12916-025-04295-5)
Supplement: Supplementary file 1 — Additional file 1: Tables S1-S11; Figures S1-S4; Supplementary Methods. Table S1 – STROBE Statement. Table S2 – ICD-9 and ICD-10 codes for dementia. Table S3 – Missing data. Table S4 – SNP information. Table S5 – Association of each instrument with all-cause dementia. Table S6 – SBP results for all models. Table S7 – DBP results for all models. Table S8 – Hypertension results for all models. Table S9 – Sensitivity analyses (SBP). Table S10 – Sensitivity analyses (DBP). Table S11 – Sensitivity analyses (hypertension). Figure S1 – Venn diagram for hypertension variable. Figure S2 – Leave-one-out analyses (SBP). Figure S3 – Leave-one-out analyses (DBP). Figure S4 – Leave-one-out analyses (hypertension). Supplementary Methods – Assessment of confounders [file 12916_2025_4295_MOESM1_ESM.docx]

**Additional file 1**

**Contents**

| **Supplementary Tables** |  |
| --- | --- |
| Table S1: STROBE Statement | Page 2-3 |
| Table S2: ICD-9 and ICD-10 codes for dementia | Page 4 |
| Table S3: Missing data | Page 5 |
| Table S4: SNP information | Page 6 |
| Table S5: Association of each instrument with all-cause dementia | Page 7 |
| Table S6: SBP(mmHg) results for all models | Page 8 |
| Table S7: DBP(mmHg) results for all models | Page 9 |
| Table S8: Hypertension results for all models | Page 10 |
| Table S9: Sensitivity analyses (SBP) | Page 11 |
| Table S10: Sensitivity analyses (DBP) | Page 12 |
| Table S11: Sensitivity analyses (hypertension) | Page 13 |
| **Supplementary Figures** |  |
| Figure S1: Venn diagram for hypertension variable | Page 14 |
| Figure S2: Leave-one-out analyses (SBP) | Page 15 |
| Figure S3: Leave-one-out analyses (DBP) | Page 16 |
| Figure S4: Leave-one-out analyses (hypertension) | Page 17 |
| **Supplementary Methods** |  |
| Assessment of Confounders | Page 18 |

**Supplementary Tables**

**Table S1:** STROBE Statement—Checklist of items that should be included in reports of ***cohort studies***

|  | | Item No | Recommendation | Page No |
| --- | --- | --- | --- | --- |
| **Title and abstract** | | 1 | (*a*) Indicate the study’s design with a commonly used term in the title or the abstract | 1 |
|  |  |  | (*b*) Provide in the abstract an informative and balanced summary of what was done and what was found | 2-3 |
| Introduction | | | | |
| Background/rationale | | 2 | Explain the scientific background and rationale for the investigation being reported | 4-6 |
| Objectives | | 3 | State specific objectives, including any prespecified hypotheses | 6-8 |
| Methods | | | | |
| Study design | | 4 | Present key elements of study design early in the paper | 8-10 |
| Setting | | 5 | Describe the setting, locations, and relevant dates, including periods of recruitment, exposure, follow-up, and data collection | 8 |
| Participants | | 6 | (*a*) Give the eligibility criteria, and the sources and methods of selection of participants. Describe methods of follow-up | 8 |
|  |  |  | (*b*) For matched studies, give matching criteria and number of exposed and unexposed | N/A |
| Variables | | 7 | Clearly define all outcomes, exposures, predictors, potential confounders, and effect modifiers. Give diagnostic criteria, if applicable | 9-13 |
| Data sources/ measurement | | 8* | For each variable of interest, give sources of data and details of methods of assessment (measurement). Describe comparability of assessment methods if there is more than one group | 9-13 |
| Bias | | 9 | Describe any efforts to address potential sources of bias | 12-14&Tab1&Fig1 |
| Study size | | 10 | Explain how the study size was arrived at | 8-9 |
| Quantitative variables | | 11 | Explain how quantitative variables were handled in the analyses. If applicable, describe which groupings were chosen and why | 9-12&Supp1 |
| Statistical methods | | 12 | (*a*) Describe all statistical methods, including those used to control for confounding | 12-14&SuppMethods |
|  |  |  | (*b*) Describe any methods used to examine subgroups and interactions | N/A |
|  |  |  | (*c*) Explain how missing data were addressed | 9&Tab2 |
|  |  |  | (*d*) If applicable, explain how loss to follow-up was addressed | N/A |
|  |  |  | (*e*) Describe any sensitivity analyses | 14 |
| Results | | | |  |
| Participants | | 13* | (a) Report numbers of individuals at each stage of study—eg numbers potentially eligible, examined for eligibility, confirmed eligible, included in the study, completing follow-up, and analysed | 14&Tab2 |
|  |  |  | (b) Give reasons for non-participation at each stage | N/A |
|  |  |  | (c) Consider use of a flow diagram | N/A |
| Descriptive data | | 14* | (a) Give characteristics of study participants (eg demographic, clinical, social) and information on exposures and potential confounders | Tab2 |
|  |  |  | (b) Indicate number of participants with missing data for each variable of interest | Tab2&SuppTab2 |
|  |  |  | (c) Summarise follow-up time (eg, average and total amount) | N/A |
| Outcome data | | 15* | Report numbers of outcome events or summary measures over time | N/A |
| Main results | 16 | (*a*) Give unadjusted estimates and, if applicable, confounder-adjusted estimates and their precision (eg, 95% confidence interval). Make clear which confounders were adjusted for and why they were included | | 15&Fig2-4; SuppTab4-9 |
|  |  | (*b*) Report category boundaries when continuous variables were categorized | | Supp methods |
|  |  | (*c*) If relevant, consider translating estimates of relative risk into absolute risk for a meaningful time period | | N/A |
| Other analyses | 17 | Report other analyses done—eg analyses of subgroups and interactions, and sensitivity analyses | | 16 |
| Discussion | | | | |
| Key results | 18 | Summarise key results with reference to study objectives | | 15&17 |
| Limitations | 19 | Discuss limitations of the study, taking into account sources of potential bias or imprecision. Discuss both direction and magnitude of any potential bias | | 21-25 |
| Interpretation | 20 | Give a cautious overall interpretation of results considering objectives, limitations, multiplicity of analyses, results from similar studies, and other relevant evidence | | 17-18 |
| Generalisability | 21 | Discuss the generalisability (external validity) of the study results | | 18-20 |
| Other information | | | | |
| Funding | 22 | Give the source of funding and the role of the funders for the present study and, if applicable, for the original study on which the present article is based | | 28 |

*Give information separately for exposed and unexposed groups.

**Table S2:** ICD-9 and ICD-10 codes to extract from Primary Care records and Hospital Episode Statistics

| ICD-9 dementia codes | ICD-10 dementia codes |
| --- | --- |
| 290.2, 290.3, 290.4, 291.2, 294.1, 331.0, 331.1, 331.2, 331.5 | A81.0, F00, F00.0, F00.1, F00.2, F00.9, F01, F01.0, F01.1, F01.2, F01.3, F01.8, F01.9, F02, F02.0, F02.1, F02.2, F02.3, F02.4, F02.8, F03, F05.1, F10.6, G30, G30.0, G30.1, G30.8, G30.9, G31.0, G31.1, G31.8, I67.3 |

**Table S3:** Missing data

| **Outcome** | **SBP** | | | **DBP** | | | **Hypertension** | | |
| --- | --- | --- | --- | --- | --- | --- | --- | --- | --- |
|  | **n** | **missing** | **% missing** | **n** | **missing** | **% missing** | **n** | **missing** | **% missing** |
| following exclusions, missing parental score and SBP | 445,167 |  |  | 445,168 |  |  | 445,911 |  |  |
| **covariate added** |  |  |  |  |  |  |  |  |  |
| age | 445,167 | 0 | **0.00%** | 445,168 | 0 | **0.00%** | 445,911 | 0 | **0.00%** |
| ses | 444,627 | 540 | **0.12%** | 444,628 | 540 | **0.12%** | 445,368 | 543 | **0.12%** |
| edu(qualifications) | 438,099 | 6,528 | **1.49%** | 438,100 | 6,528 | **1.49%** | 438,800 | 6,568 | **1.50%** |
| ethnicity | 436,845 | 1,254 | **0.29%** | 436,846 | 1,254 | **0.29%** | 437,538 | 1,262 | **0.29%** |
| smoking | 435,505 | 1,340 | **0.31%** | 435,506 | 1,340 | **0.31%** | 436,195 | 1,343 | **0.31%** |
| bmi | 434,019 | 1,486 | **0.34%** | 434,020 | 1,486 | **0.34%** | 434,371 | 1,824 | **0.42%** |
| salt | 433,994 | 25 | **0.01%** | 433,995 | 25 | **0.01%** | 434,346 | 25 | **0.01%** |
| alcohol | 433,764 | 230 | **0.05%** | 433,765 | 230 | **0.05%** | 434,115 | 231 | **0.05%** |
| exercise | 433,764 | 0 | **0.00%** | 433,765 | 0 | **0.00%** | 434,115 | 0 | **0.00%** |
|  |  |  |  |  |  |  |  |  |  |
|  |  |  |  |  |  |  |  |  |  |
|  |  |  |  |  |  |  |  |  |  |

**Table S4:** SNP exposure (Alzheimer’s disease) information for the 32 included SNPs

| SNP | Effect_Allele | Other_Allele | Beta | SE | P |
| --- | --- | --- | --- | --- | --- |
| rs111278137 | A | G | -0.4735 | 0.0713 | 3.12E-11 |
| rs11257242 | G | C | 0.0841 | 0.0154 | 4.73E-08 |
| rs114812713 | C | G | 0.2980 | 0.0431 | 4.71E-12 |
| rs11668327 | C | G | -0.4126 | 0.0223 | 1.98E-76 |
| rs11669005 | A | G | 0.1085 | 0.0164 | 3.69E-11 |
| rs117316645 | A | G | 0.2709 | 0.0349 | 8.35E-15 |
| rs11767557 | C | T | -0.1028 | 0.0182 | 1.62E-08 |
| rs12151021 | G | A | -0.1071 | 0.0169 | 2.34E-10 |
| rs12590654 | A | G | -0.0906 | 0.0157 | 7.89E-09 |
| rs138607350 | G | T | 1.0175 | 0.0651 | 4.56E-55 |
| rs144645090 | A | G | 0.3633 | 0.0533 | 9.35E-12 |
| rs147711004 | A | G | 1.1354 | 0.0366 | 2.73E-211 |
| rs1582763 | A | G | -0.1232 | 0.0149 | 1.36E-16 |
| rs2965112 | G | A | -0.2120 | 0.0261 | 4.56E-16 |
| rs2965169 | C | A | -0.2056 | 0.0161 | 2.41E-37 |
| rs3112439 | C | G | -0.2677 | 0.0215 | 1.38E-35 |
| rs34665982 | C | T | -0.0967 | 0.0166 | 5.70E-09 |
| rs3740688 | T | G | 0.0935 | 0.0144 | 8.41E-11 |
| rs3851179 | C | T | 0.1198 | 0.0148 | 5.75E-16 |
| rs4802241 | C | A | -0.1460 | 0.0211 | 4.53E-12 |
| rs62117224 | A | G | -0.1773 | 0.0188 | 4.07E-21 |
| rs6710467 | A | G | 0.1330 | 0.0195 | 9.07E-12 |
| rs6733839 | T | C | 0.1693 | 0.0154 | 4.11E-28 |
| rs679515 | C | T | -0.1508 | 0.0183 | 1.72E-16 |
| rs72654437 | A | G | -0.2929 | 0.0490 | 2.27E-09 |
| rs72654445 | A | G | -0.5425 | 0.0811 | 2.24E-11 |
| rs73223431 | T | C | 0.0936 | 0.0153 | 9.50E-10 |
| rs75463276 | G | C | 0.4064 | 0.0618 | 4.83E-11 |
| rs77301115 | A | G | 0.9486 | 0.0427 | 2.43E-109 |
| rs8100183 | T | C | 0.1261 | 0.0202 | 4.30E-10 |
| rs867230 | A | C | 0.1333 | 0.0158 | 3.26E-17 |
| rs9381563 | T | C | -0.0821 | 0.0148 | 2.90E-08 |

**Table S5:** Association of each instrument with participant all-cause dementia (prevalent or through linked primary care or hospital episode statistics for small subset)

|  | N | N with dementia | OR | 95% CI(OR) | p-value |
| --- | --- | --- | --- | --- | --- |
| Parental dementia instrument score vs all_cause_dementia | 446,790 | 2,278 | 3.2281 | 2.5972 to 4.0123 | <0.0001 |
| Participant genetic instrument score vs all_cause_dementia | 462,885 | 2,541 | 1.6156 | 1.5350 to 1.7003 | <0.0001 |

**Table S6:** SBP(mmHg) results for all models

| Model | Mean | 95% CI | p-value | Model | Mean | 95% CI | p-value |
| --- | --- | --- | --- | --- | --- | --- | --- |
| PDIS analyses (complete cases n = 433764) | | | | **PGIS analyses (complete cases n = 330643)** | | | |
| 1. Crude | -0.240 | -0.03 to -0.19 | 0.0176 | 1. Crude | 0.022 | -0.04 to 0.09 | 0.4901 |
| 2. Crude with BP correction | -0.223 | 0.28 to -0.16 | 0.0176 | 2. Crude with BP correction | 0.042 | -0.03 to 0.11 | 0.2381 |
| 3. Age-adjusted | 0.074 | 0.02 to 0.13 | 0.0052 | 3. Age- Sex- and Genetic principal components-  adjusted | 0.042 | -0.02 to 0.10 | 0.1682 |
| 4. Age-adjusted with BP correction | 0.179 | 0.12 to 0.24 | <0.0001 | 4. Age- Sex- and Genetic principal components-  adjusted with BP correction (*main model*) | 0.068 | 0.00 to 0.13 | 0.0366 |
| 5. Full confounder-adjusted | 0.040 | -0.01 to 0.09 | 0.1177 | 5. Full confounder-adjusted | 0.065 | 0.01 to 0.12 | 0.0273 |
| 6. Full confounder-adjusted with  BP correction (*main model*) | 0.116 | 0.06 to 0.17 | <0.0001 | 6. Full confounder-adjusted with BP correction | 0.107 | 0.05 to 0.17 | 0.0007 |

PDIS = Parental dementia instrument score; PGIS = Participant genetic instrument score

**Table S7:** DBP(mmHg) results for all models

| Model | Mean | 95% CI | p-value | Model | Mean | 95% CI | p-value |
| --- | --- | --- | --- | --- | --- | --- | --- |
| PDIS analyses (complete cases n = 433765) | | | | **PGIS analyses (complete cases n = 330644)** | | | |
| 1. Crude | -0.013 | -0.04 to 0.02 | 0.0176 | 1. Crude | -0.017 | -0.05 to 0.02 | 0.3431 |
| 2. Crude with BP correction | -0.002 | -0.03 to 0.03 | 0.0176 | 2. Crude with BP correction | -0.003 | -0.04 to 0.03 | 0.862 |
| 3. Age-adjusted | 0.009 | -0.02 to 0.04 | 0.5493 | 3. Age- Sex- and Genetic principal components-  adjusted | -0.019 | -0.05 to 0.01 | 0.2679 |
| 4. Age-adjusted with BP correction | 0.079 | 0.05 to 0.11 | <0.0001 | 4. Age- Sex- and Genetic principal components-  adjusted with BP correction (*main model*) | -0.001 | -0.04 to 0.04 | 0.9418 |
| 5. Full confounder-adjusted | -0.019 | -0.05 to 0.01 | 0.1931 | 5. Full confounder-adjusted | 0.008 | -0.02 to 0.04 | 0.6447 |
| 6. Full confounder-adjusted with  BP correction (*main model*) | 0.032 | 0.00 to 0.06 | 0.0419 | 6. Full confounder-adjusted with BP correction | 0.036 | 0.00 to 0.07 | 0.0443 |

PDIS = Parental dementia instrument score; PGIS = Participant genetic instrument score

**Table S8:** Hypertension results for all models

| Model | OR | 95% CI | p-value | Model | Mean | 95% CI | p-value |
| --- | --- | --- | --- | --- | --- | --- | --- |
| PDIS analyses (complete cases n = 434115) | | | | **PGIS analyses (complete cases n = 330886)** | | | |
| 1. Crude | 0.993 | 0.99 to 1.00 | 0.0176 | 1. Crude | 1.002 | 1.00 to 1.01 | 0.5317 |
| 2. Age-adjusted | 1.029 | 1.02 to 1.04 | <0.0001 | 2. Age-adjusted | 1.005 | 1.00 to 1.01 | 0.1823 |
| 3. Full confounder-adjusted (*main model*) | 1.021 | 1.01 to 1.03 | <0.0001 | 3. Full confounder-adjusted (*main model*) | 1.011 | 1.00 to 1.02 | 0.0035 |

PDIS = Parental dementia instrument score; PGIS = Participant genetic instrument score

**Table S9:** Sensitivity analyses including additional potential confounders in the association between parental dementia instrument score and SBP

| *SBP(mmHg)* |  |  |  |  |
| --- | --- | --- | --- | --- |
| Model | **n** | **beta** | **95% CI** | **p** |
| Main model (Full confounder corrected with BP correction) | 433764 | 0.1161 | 0.0621 to 0.1701 | <0.0001 |
| Main model on participants with data on HDLc, LDLc, Totalc, Triglycerides, C-reactive P and HcA1c | 356248 | 0.0889 | 0.0294 to 0.1483 | 0.0034 |
| With addition of HDLc, LDLc, Totalc, Triglycerides, C-reactive P, HcA1c | 356248 | 0.0985 | 0.0394 to 0.1576 | 0.0011 |

**Table S10:** Sensitivity analyses including additional potential confounders in the association between parental dementia instrument score and DBP

| *DBP(mmHg)* |  |  |  |  |
| --- | --- | --- | --- | --- |
| Model | **n** | **beta** | **95% CI** | **p** |
| Main model (Full confounder corrected with BP correction) | 433765 | 0.0317 | 0.0012 to 0.0622 | 0.0418 |
| Main model on participants with data on HDLc, LDLc, Totalc, Triglycerides, C-reactive P and HcA1c | 356249 | 0.0144 | -0.0192 to 0.0479 | 0.4019 |
| With addition of HDLc, LDLc, Totalc, Triglycerides, C-reactive P, HcA1c | 356249 | 0.0149 | -0.0185 to 0.0484 | 0.381 |

**Table S11:** Sensitivity analyses including additional potential confounders in the association between parental dementia instrument score and hypertension

| *Hypertension* |  |  |  |  |
| --- | --- | --- | --- | --- |
| Model | **n** | **OR** | **95% CI** | **p** |
| Main model (Full confounder corrected with BP correction) | 434115 | 1.0209 | 1.0143 to 1.0275 | <0.0001 |
| Main model on participants with data on HDLc, LDLc, Totalc, Triglycerides, C-reactive P and HcA1c | 356249 | 1.0175 | 1.0102 to 1.0247 | <0.0001 |
| With addition of HDLc, LDLc, Totalc, Triglycerides, C-reactive P, HcA1c | 356519 | 1.0189 | 1.0116 to 1.0262 | <0.0001 |

**Supplementary Figures**


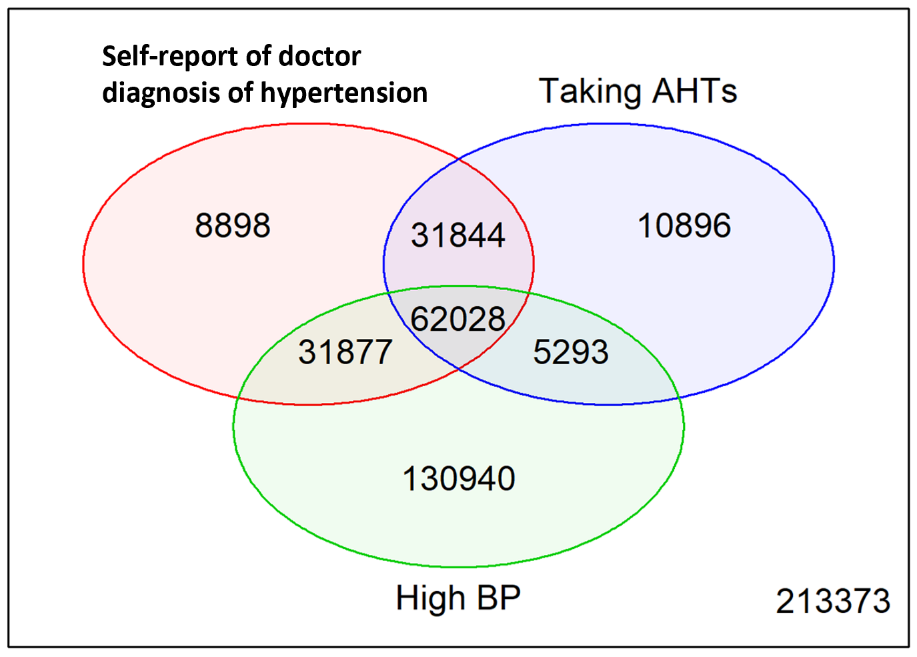


**Figure S1:** Venn diagram showing composition of Hypertension variable


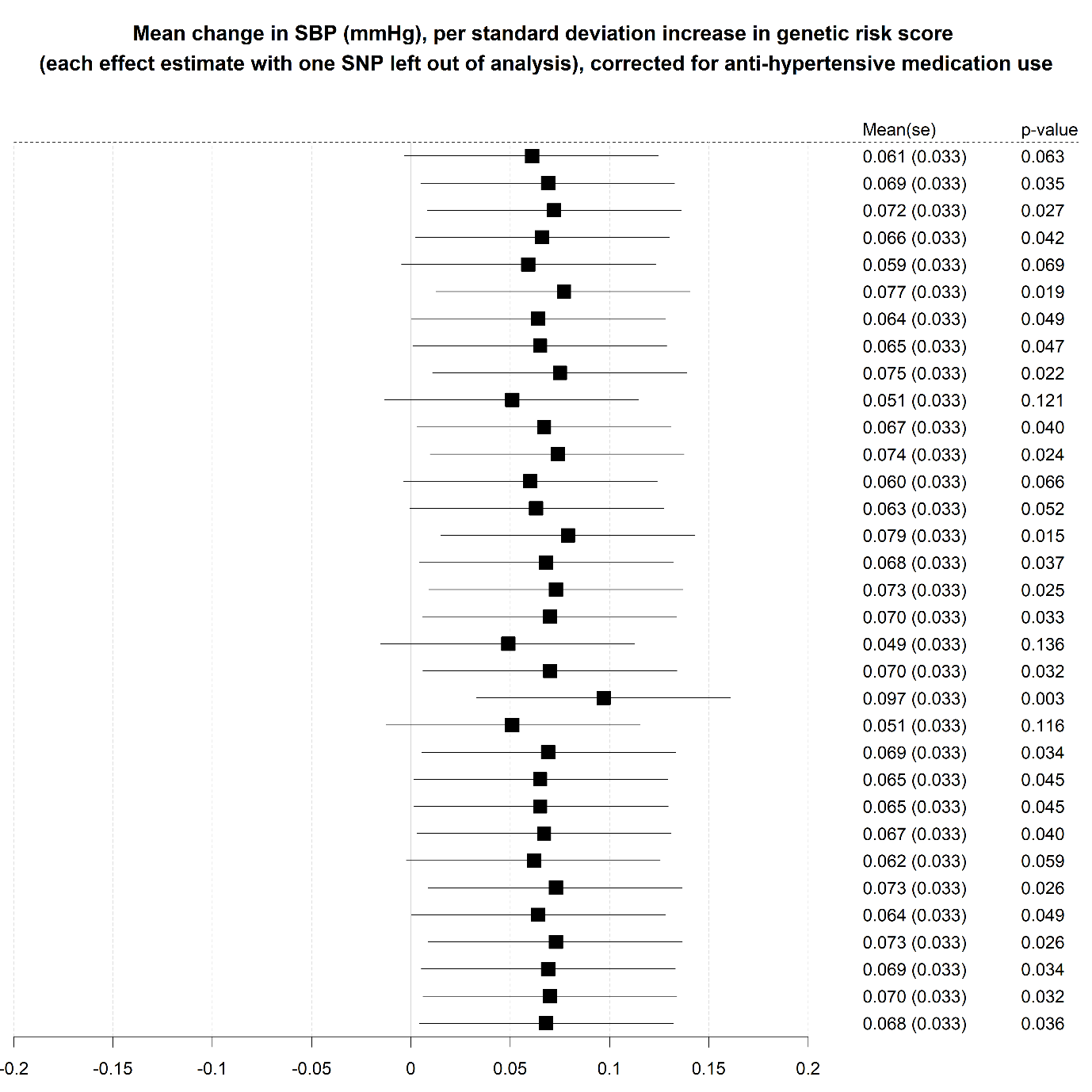


**Figure S2:** Leave one out analyses of SNPs in the PGIS to assess potential for influential outliers in analysis of SBP


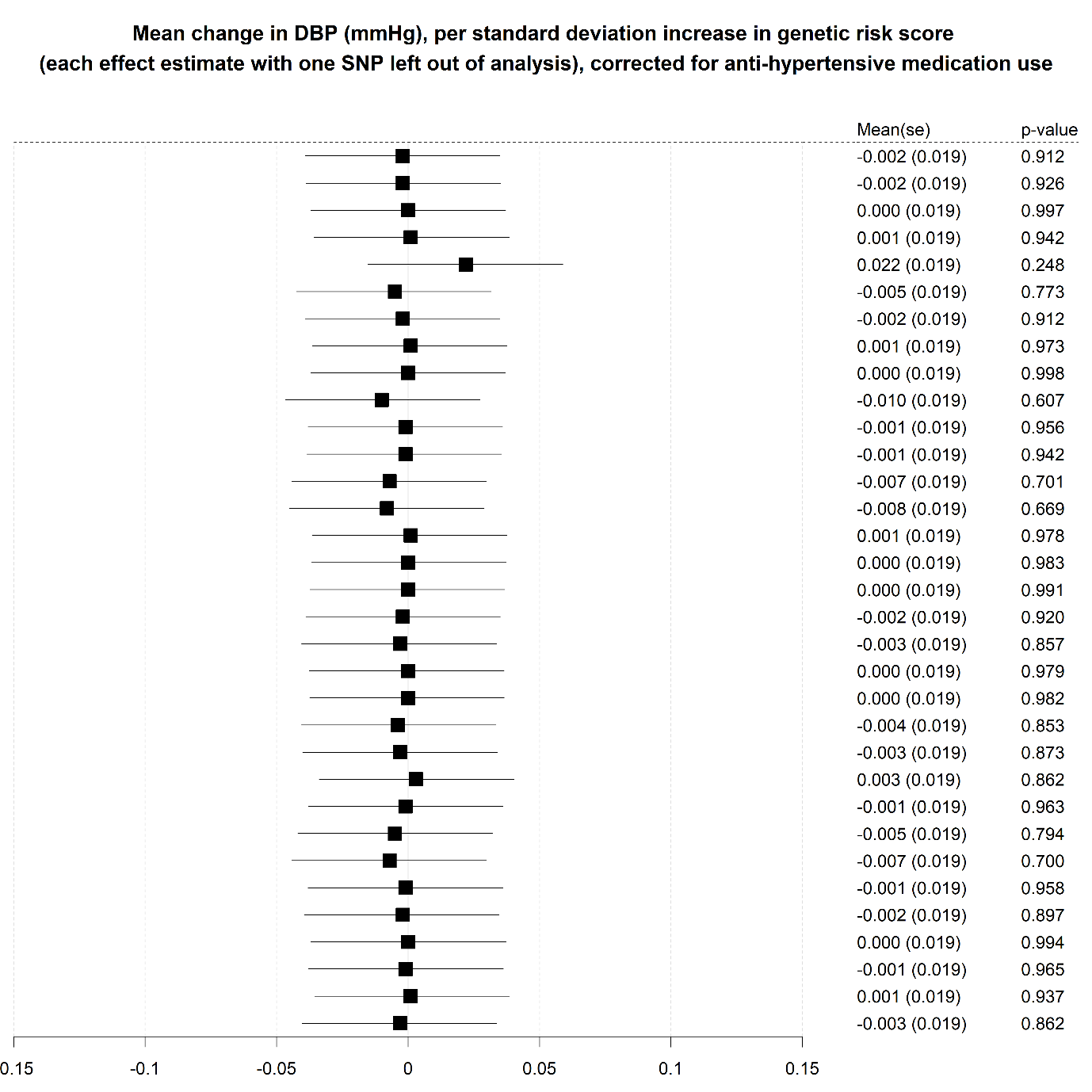


**Figure S3:** Leave one out analyses of SNPs in the PGIS to assess potential for influential outliers in analysis of DBP


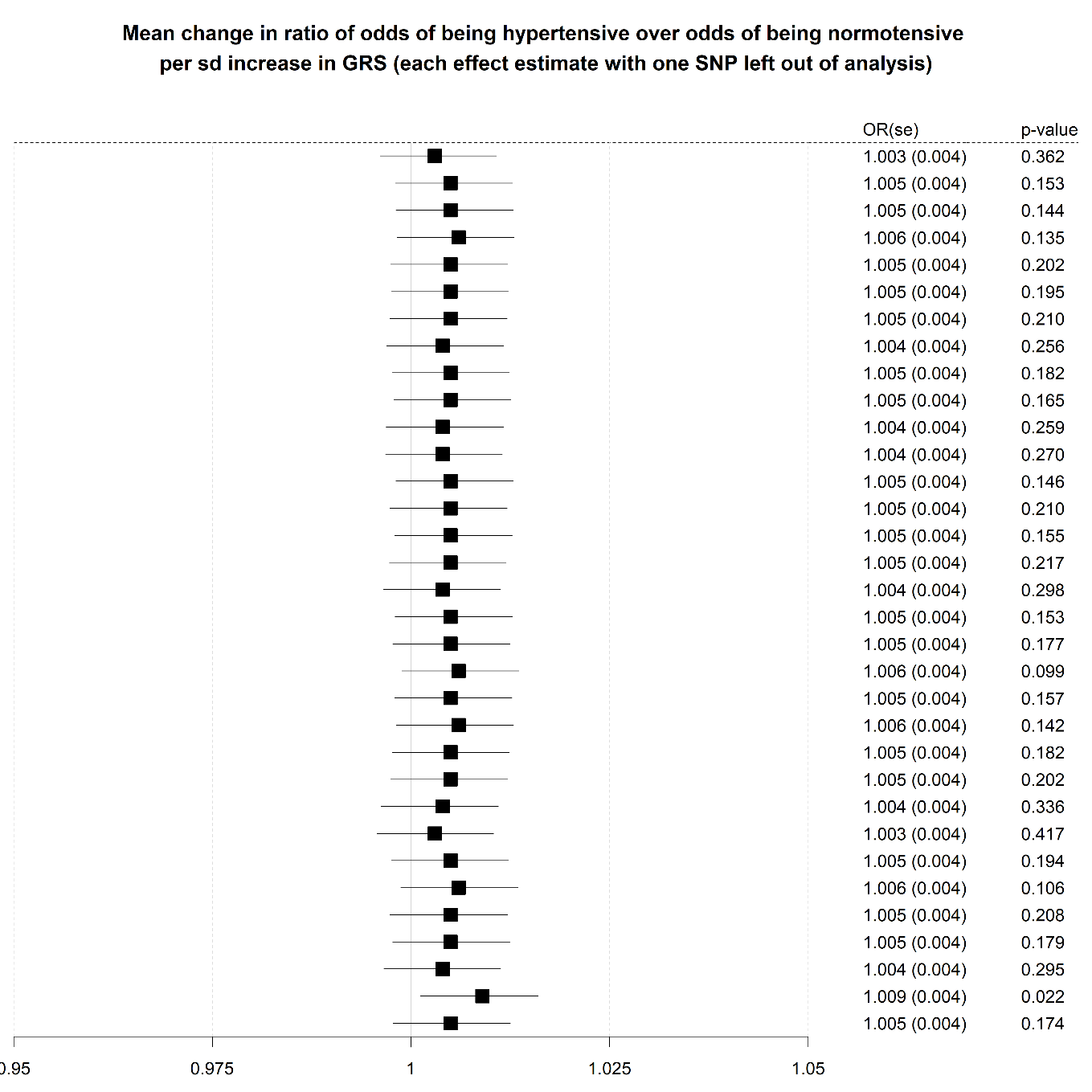


**Figure S4:** Leave one out analyses of SNPs in the PGIS to assess potential for influential outliers in analysis of hypertension

**Supplementary Methods**

**Assessment of confounders**

All confounders were measured as part of participants’ in-person assessment and were decided *a priori* from UK Biobank data showcase, before having access to the data. If missing from baseline assessment, we replaced with that recorded at later assessments. If missing from all assessments, we marked as missing. We used a continuous age variable and a binary sex variable. Due to a large amount of missing data for the age participants left education, we used participant’s highest qualification as a measure of education, and categorised into three ordered categories: (1) CSE/O-Level/GCSE or equivalent (~43%); (2) NVQ/HND/HNC/A-levels/AS levels/Other professional qualification or equivalent (~23%); (3) College/University degree or equivalent (~32%). There were 20 categories available for ethnicity, many with sparse data, so we combined into a binary variable of ‘white’ (white, British, Irish, any other white background; ~94%) and ‘aggregated ethnic minority groups’ (African, Asian or Asian British, Bangladeshi, black or black British, Caribbean, Chinese, Indian, mixed, Pakistani, white and Asian, white and black African, white and black Caribbean, any other Asian background, any other black background, any other mixed background, other ethnic group; ~5%). We combined categories of smoking into never (~55%), previous (~35%) and current (~11%) smokers. We kept body mass index (BMI) as a continuous variable. For physical activity we grouped into: no days (~35%); 1-2 days (28%); or 3 or more days (36%) per week of >10 minutes of exercise. We used ‘preference for adding salt to food’ categorised into: never/rarely (~55%); sometimes (~28%); usually (~12%) and always (~5%) as a proxy measure of dietary salt intake. We categorised alcohol into 6 ordered categories of use: never (~8%); special occasions only (~12%); 1-3 times per month (~11%); 1-2 times per week (~26%); 3-4 times per week (23%) and daily/almost daily (~20%). Finally, we used the Townsend deprivation index as a measure of socio-economic position.
